# Testing the acceptability and feasibility of video observational methodology to measure parent-adolescent communication and interaction

**DOI:** 10.3389/frcha.2023.1122841

**Published:** 2023-08-24

**Authors:** Fortunate Lekhuleni, Rachana Desai, Bronwyne Coetzee, Rebecca Pearson, Tamsen Jean Rochat

**Affiliations:** ^1^ DSI-NRF Centre of Excellence in Human Development, University of the Witwatersrand, Johannesburg, South Africa; ^2^ Department of Psychology, Stellenbosch University, Stellenbosch, South Africa; ^3^ Department of Psychology, Manchester Metropolitan University, Manchester, United Kingdom

**Keywords:** acceptability, feasibility, video observation, parent-adolescent, communication, interaction

## Abstract

**Background:**

Existing research has shown that the parent-adolescent relationship and its associated communication and interaction styles are important for adolescent development and outcomes. Measuring parent-adolescent communication and interaction using self-report methods has substantial research limitations. Video observational methodologies offer a novel and more objective approach to measuring parent-adolescent communication and interaction from the point of view of participants. This study aims to explore the feasibility and acceptability of this methodology, and analysis using automated coding software in an urbanized context.

**Methods:**

This study recruited parent-adolescent pairs in Soweto, South Africa which included 11–15-year-old adolescents and their biological parents. Parent-adolescent communication and interactions were measured using novel video observational portable head cameras called “Teencams”. Feasibility was evaluated by testing three observational game tasks (Matching pairs card game, Jenga and Charades) to stimulate communication and interaction between 16 parent-adolescent pairs, and the Teencam's ability to record video and audio content. Acceptability was explored using one-on-one interviews with the parents (*n* = 14), on whether they found the Teencam comfortable to wear, whether the parents believed their adolescents acted naturally, and which observational game tasks were feasible during their interactions. The videos were analysed using automated coding software called FaceReader which detects and codes basic facial expressions.

**Results:**

The Teencam methodology was found to be feasible and acceptable amongst parent-adolescent pairs in Soweto, South Africa. The Matching pairs card game stimulated excellent interaction and communication with good video and audio quality. Some feasibility limitations were identified in the operations (switching on/off and starting recording), the ability of the device to cope with the movement of the participants, and the lighting conditions of the room, all of which resulted in poor coding and analytic output from FaceReader. Refinements and adjustments were made to the methodological protocol by improving the head cameras and lighting conditions and refining the Matching pairs card game, which resulted in improved analytic output from FaceReader.

**Conclusion:**

Based on these findings, a methodological protocol was developed to measure parent-adolescent interaction and communication in an urban setting. The unique contribution of this research lies in its potential to lead to improved methodologies for measuring parent-adolescent communication and interactions.

## 1. Introduction

Parenting and the quality of the parent-child relationship are important for adolescent health and well-being. Numerous studies, including research in South Africa, have illustrated associations between the parent-child relationship and positive outcomes for adolescents, especially in the early transition into adolescence (1, 2). The parent-adolescent relationship is however often strained as a consequence of developmental shifts involved as adolescents move towards autonomy (3). Adolescents in South Africa also face a myriad of challenges: poor educational outcomes, various forms of poverty and abuse, and less-than-ideal mental and physical health, including high rates of HIV (4). In high-risk environments, it may be especially important to understand and facilitate relationship support for parents and adolescents that ensure adolescents can grow towards autonomy and independence while managing potential risks.

Positive parent-child communication influences the reduction of risky behaviours among adolescents such as substance use and abuse, delinquent behaviours, and risky sexual behaviours (5–7). The quality of the parent-adolescent relationship is reflected in parent-adolescent communication and interaction. Parent-adolescent communication is a process through which beliefs, attitudes, values, expectations and knowledge are conveyed between parents and adolescents (8). Parent-child interactions are rooted in daily activities and function as an enhancement to the parent-child relationship. During an interaction with a parent, children learn social skills such as sharing, cooperating, and respecting others’ belongings (9). While patterns of parent-child communication and interactions begin to develop earlier in childhood, during adolescence, parents and adolescents face new challenges in their relationship with changing developmental needs in the adolescent, and changes in the parenting context (10, 11).

Methods for studying these relationships have traditionally used self-reported questionnaires, which have many limitations such as systematic biases (12) and may be susceptible to misinterpretation of the actual interactions between parents and adolescents. Furthermore, concordance between parent and adolescent reports of each other's emotions and behaviours tends to be low (13), all of which raises substantial questions about the validity of these self-report measures. In addition, some research has shown that when parent self-reports are compared to directly observed objective measures of parenting, most parents report their parenting to be worse than it is (14). Compared to the measurement issues involved in self-report data, ratings of observed parenting behaviours by trained researchers are considered more reliable (12, 13, 15). Yet these methods have been expensive to collect and have been very time-consuming to score and code, making them far less evident in literature from lower-income contexts.

Video observational methodologies using video observational equipment in the absence of a researcher, have been used widely for decades and have been found to have enormous benefits including objectivity, reliability, and opportunities for detailed analysis (16). However, there are challenges with video observational methods related to camera reactivity in which children get distracted by the cameras and participants, in general, change their behaviour because of camera awareness. While direct observations of relationships have the advantage of being objective, they also have several disadvantages in that they are costly to implement and time-consuming to code and make use of (17).

Currently, new technology has emerged called head cameras or spy cameras which are worn on the body or head to record video and audio of the participant's viewpoints, behaviours and environment in a more naturalistic way (18, 19). The advantages of these wearable cameras include the low cost of the equipment, the elimination of a researcher being present, reducing potential influences of the researcher on parent-adolescent behaviour, and that it enables the ability to record and sync the viewpoints of the parent and their adolescent child, so different perspectives, emotions and behaviours are captured under more naturalistic conditions. Alongside this, highly advanced coding software which substantially reduces the coding time of observations by automating the preparation of data clips for coding, now has the potential to strengthen methodological approaches. Two studies examining parent-adolescent interactions utilized a validated coding software known as FaceReader to analyze facial expressions and emotional states (20, 21). The footage for analysis was obtained from wall-mounted cameras. The use of this coding software may particularly be beneficial in resource-constrained contexts like South Africa, where it could minimise the time and resources required for coding and analysing objective data.

Most observational research that make use of wearable head cameras with parents and children has focused on the first five years of life (22). They have previously been used for recording infants’ eye views of their environment and infant-mother interactions (18, 23, 24). Almost no research to date has developed developmentally standardised methodologies for observing parent-adolescent communication and interaction in Low- and middle-income countries (LMIC). Less is understood about the validity of parent-adolescent observational protocols and the acceptability or feasibility of this approach in these settings. Little is also known about how to code these data within the South African cultural and socio-economic context.

The current study (made up of two studies) piloted a method to measure parent-adolescent communication and interaction in a low-income urbanized setting characterized by a diverse ethnic and cultural, predominantly African population. The study findings will likely inform and strengthen the development of interventions to improve parent-adolescent communication and interaction.

The primary aims of each study was as follows:

Study 1:
  1. To pilot test the feasibility of three observational game tasks that elicit prosocial behavior, competitiveness, problem-solving, conflict resolution, and communication skills between the parent-adolescent pairs while wearing head cameras, which records audio and video footage.
  2. To explore the acceptability of the video observational methodology based on parent feedback from individual interviews.
Study 2:
  3. To explore the ability of automated coding software to capture and analyse facial expressions and emotional cues to inform the development of a methodological protocol.

## 2. Methods

### 2.1. Research setting

The research took place at the MRC Developmental Pathways to Health Research Unit (DPHRU) research site in Soweto, a low-income urban setting in South Africa. Soweto is the most populous urban residential area in South Africa, with 1.2 million residents living in 200 square kilometres (6,357 per km^2^) an estimated 300,000 of whom are adolescents aged 11–18 years. Soweto faces challenges common to highly urban metropolitan areas in LMIC including poor housing, overcrowding, high unemployment and poor infrastructure. This research was embedded in an existing study called the BEACON study. The BEACON study is a large-scale cohort study of parents and young adolescents which aims to test the role of executive function on adolescent risk behaviours. Within the BEACON study, there exists a subgroup known as the BEACON Advisory Group (BAG), consisting of parent and adolescent pairs. The primary purpose of this group is to pilot test the methodologies and measures that will later be implemented with the larger BEACON cohort.

### 2.2. Inclusion and exclusion criteria

The inclusion criteria across study 1 and 2 were that adolescents be aged between 11 and 15 years, that a biological parent who is also a primary caregiver take part, a parent’s willingness to participate and an adolescent’s willingness to assent, the absence of mental and physical disabilities which may hinder participation, and residency in the study area as well as the intention to remain in the study area for 3 years.

### 2.3. Ethical considerations

Ethical approval (protocol number: M190801) was granted by the University of the Witwatersrand Ethics Committee in the Health Sciences Faculty for both studies and associated phases. Parental consent and adolescent assent were obtained for participation in the study. Participants were informed about the voluntary nature of their involvement and their right to withdraw from any activities at any time without consequence. To ensure confidentiality, transcripts and video data were assigned Participant Identification Numbers (PIDs) and stored securely on a password-protected computer.

## 3. Study 1: feasibility of the three observational game tasks (Phase 1)

Study 1 addresses objective 1 and 2 of this study and consisted of two phases. The first phase explored the feasibility of the three observational game tasks. The second phase explored the acceptability of the Teencam methodology.

### 3.1. Research participants

The participants for this phase of the study consisted of a BEACON advisory group (BAG). BAG participants were recruited from two sources: key informant referrals and enumeration lists. Trained research assistants were responsible for identifying and approaching up to five potential parent-adolescent participants through door-to-door recruitment. These initial participants were also requested to refer others they may know who could be interested in joining the study. Additionally, enumeration lists consisting of participant contact details were obtained from other cohort studies conducted in the unit. These lists contained individuals who had previously given consent to be contacted for participation in other studies and were purposefully selected and invited to take part in the BAG study. Pairs were consecutively approached and 16 parent-adolescent pairs were recruited and had given consent and assent to be BAG members.

### 3.2. Materials

#### 3.2.1. Teencams

**Figure 1:**
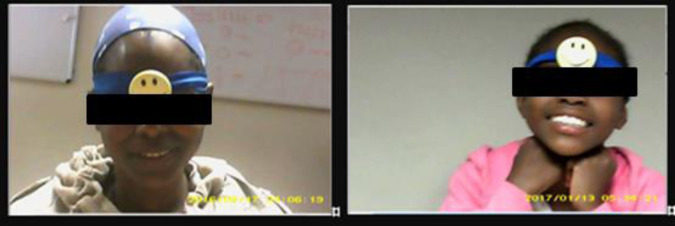
Parent and adolescent view of the Teencam footage.

The head cameras called Teencams used to test the feasibility of the observational task were novelty spy cameras in the form of lapel badges and were yellow with a black smiley face. They were head-worn by attaching them onto an elastic headband (see Figure 1). The Teencams can record audio and video and store the footage on a built-in SD card. These Teencams were used to record observational tasks conducted with the BAG participants.

#### 3.2.2. Stimuli material

The stimuli materials included three different games; the Matching pairs card game, Jenga and Charades. The selection of game tasks was driven by the goal of fostering prosocial behaviour, competitiveness, problem-solving abilities, conflict resolution, and effective communication skills among participants. Game tasks were selected as a strength-based approach due to their alignment with promoting family support and the joy derived from parent-child play (25). The Matching pairs card game consisted of 52 standard card packs, of which 10 Matching card pairs were selected and used for the game. The Jenga game consists of 54 rectangular blocks, of which 27 blocks were used, stacked three per layer. The “Charades game—Soweto version”, was developed by the researchers by sourcing images and words that were relatable to the Soweto population and printing them in a card format. The images and words included landmarks in Soweto such as malls, hospitals, stadiums, the Soweto towers etc., and other common foods and day-to-day activities that people in Soweto are exposed to.

### 3.3. Procedure

Observational tasks were conducted over three different workshops with parent-adolescent pairs which spanned over three different days. Details of the procedure in each workshop are presented in Table 1. The Teencam activity took place during the refreshment break of the BEACON cohort study, wherein the parent-adolescent pairs both wore the Teencams while sitting around a table facing each other to record their communication and interaction, and to ensure that facial expressions and emotional cues were captured. The Teencam task lasted approximately 15 min each. The parent-adolescent pairs were expected to participate in all three workshops.

**Table 1:** Teencam activities per workshop and activity instructions.

Workshop number	Activity	Activity Instruction
1	Matching pairs card game	• The parents themselves were instructed to switch the Teencams on and start the recording for themselves and their adolescents. • A pack of cards (Old Maid & Go Fish: designed for 11–12-year-olds, Hearts and Crazy: designed for 13–15-year-olds), was given to each pair, which included 10 Matching pair cards with different images and words on them such as animals and people demonstrating different careers and activities. • All cards were placed facing down on the table. • Each participant took a turn to turn over two cards, if they were a matching pair, they took the pair off the table into their pack. If the cards didn't match, they were turned back over to face down again. • As the turns proceeded the players had to remember where they had seen the card before that was matching. • The participant with the most matching card pairs won the game.
2	Jenga game	• The researcher switched the Teencams on to start the recording, provided instructions for participants to not touch the Teencams and then exited the room. • Each pair was given the Jenga blocks to build a tower. • The players took turns removing a block from the tower and balancing it on top, creating a taller and increasingly unstable structure as the game progressed. • The game ended when the tower fell—either completely or if any block fell from the tower (other than the block a player moves on a turn). • The player who collapses the tower loses the game. • For this study, each pair was given 27 blocks because the entire 54 blocks would not work since they had to play the game sitting down with limited movement.
3	Charades game	• The researcher switched the Teencams on to start the recording, provided instructions for participants to not touch the Teencams and then exited the room. • The charades game started with placing word and image printouts on the table facing down, the adolescent had to start the game by pulling one card and facing it to the parent and having the parent act out clues about the image or word on the card and the adolescent had to guess what they thought the clues refer to. • The player guessing the game had three chances to guess what was on the card, if not successful the player giving clues had to give them the correct answer. • Players had to alternate to give clues and guess the words or images as shown to them by the opponent. • The player who guessed the most correct answers won the game.

Across all three workshops, before the Teencam activity started, the adolescents (in a separate room from the parents) were taught the game and then asked to teach the game to their parents. During workshop 1, the responsibility of switching on the Teencams was assigned to the parents themselves. However, this approach resulted in several cameras not being activated accurately. To prevent a recurrence of this issue in workshops 2 and 3, the researchers took on the responsibility of managing the activation and deactivation of the cameras.

### 3.4. Participant exclusions and final sample

**Figure 2:**
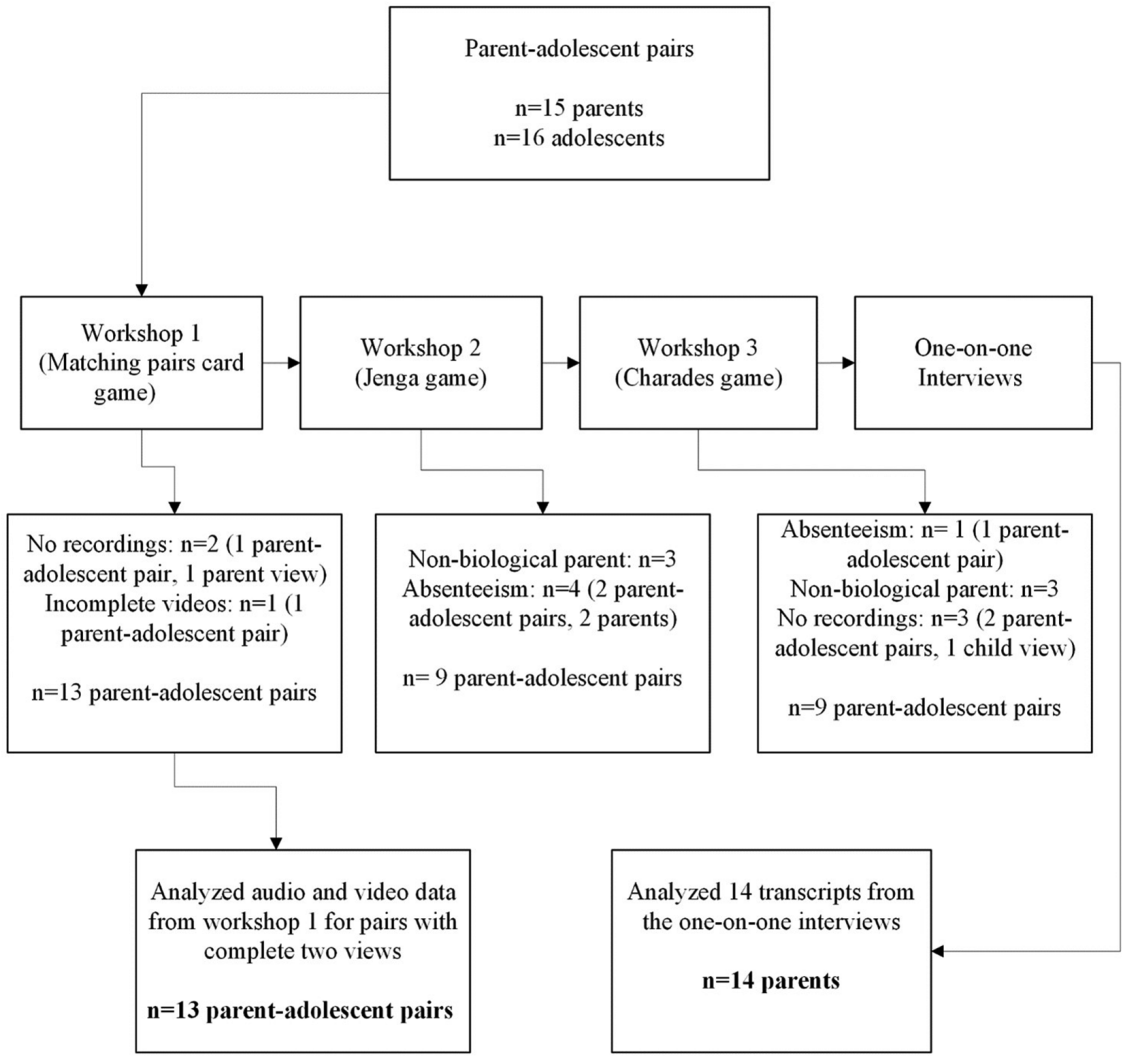
Consort diagram for study 1 of BAG participants per workshop.

As seen in Figure 2, 31 BAG members were recruited. There were 16 adolescents and 15 parents, and one parent enrolled two adolescents. In workshop 1, all parent-adolescent pairs participated but due to technical errors (cameras not recording full observations, or one/both perspectives), three pairs were therefore excluded from the study and 13 parent-adolescent pairs were included in the final sample for workshop 1. During workshop 2 and 3, an equal number of nine parent-adolescent pairs were included in the final sample. The other pairs were excluded due to absenteeism, not meeting eligibility criteria of having a biological parent or technical errors.

#### 3.4.1. Participant characteristics

In total, there were 16 parent-adolescent pairs in this pilot study as presented in Table 2. Unfortunately, one parent passed away before socio-demographic data were collected for the BAG participants via a questionaire as part of Study 2, therefore participant characteristics are only reported for 15 adolescents and 14 parents. The adolescents were predominantly female (*n* = 9, 60%) and only biological mothers were enrolled in the pilot study. The average age of the adolescents was 12.2 years (SD = 1.37) and the average age of the parents was 37.21 years (SD = 7.11). All the pairs identified themselves as African. Most of the parents were unemployed (*n* = 12, 85.71%) and over half of the parents reported having completed primary school education only.

**Table 2:** Participant characteristics of the BAG participants.

	Adolescent		Parent	
	*n*	%/mean (SD)	*n*	%/mean (SD)
Age	15	12.2 (1.37)	14	37.21 (7.11)
Gender				
Male	6	40		
Female	9	60	14	100
Race				
African			14	100
Employed			2	14.28
Highest education level				
Primary school			8	57.14
Matric (senior school certificate)			5	35.71
Post-matric			1	7.14

### 3.5. Data analysis

Videos from each workshop were manually observed and coded by the author FL, who coded each video using a yes (criteria was met)/no (criteria was not met) classification based on specified dimensions and her observational fieldnotes taken at the time of the recordings. Each of these dimensions specified below had specific criteria that were coded and counted. The coding of these dimensions was cross-checked by authors TR, BC and RD to ensure accuracy and consistency.

#### 3.5.1. Technical reliability

The Teencam's ability to successfully record interactions. The number of audio and video recordings obtained and those with technical problems were counted.

#### 3.5.2. Usability

The ability for the Teencam to be switched on and off, start and stop recording, which were obtained from researcher fieldnotes.

#### 3.5.3. Audio quality

The extent to which the researcher could hear the vocalizations articulated by the participants from the video footage and be able to transcribe them verbatim, measured using a yes/no classification system and researcher fieldnotes.

#### 3.5.4. Video quality

The ability for the researcher to detect facial expressions, eye gaze, and general facial and body movements and responses which were obtained from researcher fieldnotes.

#### 3.5.5. Feasibility of the observational task

This was determined by the participant’s ability to understand and carry out the rules of the game, and for the game to elicit communication and interaction obtained from researcher fieldnotes.

#### 3.5.6. Quality of the interaction

The extent to which the game elicited conversation and engagement between the parent-adolescent pairs obtained from researcher fieldnotes.

## 4. Results

### 4.1. Technical reliability

Technical reliability was assessed in each workshop for all participating pairs, irrespective of eligibility. The number of audio and video recordings and those with technical problems were counted. In Workshop 1, out of 16 parent-adolescent pairs, only one pair experienced recording issues, resulting in missing audio and video data. Additionally, one parent viewpoint did not record video, but audio was obtained from the other viewpoint. Thus, audio recordings were available for 15 pairs (94%), and 13 pairs had complete video and audio recordings from both parent and adolescent viewpoints (81%). In Workshop 2, no technical errors were detected among the 12 participating pairs. Workshop 3 involved 15 pairs, but two pairs had missing video and audio data. Similarly, one adolescent viewpoint lacked video recording, but audio was obtained from the other viewpoint. Consequently, Workshop 3 had audio recordings for 14 pairs (94%), and 12 pairs had complete video and audio recordings from both parent and adolescent viewpoints (80%). Overall, Teencam demonstrated excellent technical reliability, recording the different games with an overall reliability exceeding 80%.

### 4.2. Usability of the Teencam

The device had several features that impeded user functionality for the researchers. Researcher fieldnotes indicated that the Teencam devices lacked clear indicators of recording status and did not have easily identifiable start and stop buttons. Consequently, researchers had to rely on a trial-and-error approach, pressing various combinations of buttons to turn the device on or off and initiate or terminate recording. This erratic button pressing led to technical glitches, including missed session recordings. Identifying these missed recordings was only possible during video footage screening after a session when the Teencam was connected to a laptop.

### 4.3. Audio quality

The Teencam device demonstrated good speech recording capabilities. The presence of background noise generated by other participants in the same room while playing the game would sometimes hinder the clarity of the recorded conversations, posing some challenges for accurate transcription. Specifically, during the Charades game, there was a higher incidence of participants talking over each other and shouting, in contrast to the Matching pairs card game and Jenga. This discrepancy can be attributed to the forced turn-taking structure of the latter games, which facilitated smoother communication by ensuring participants did not speak over one another.

### 4.4. Video quality

As seen in Figure 1, video clips of parent-adolescent interactions capture the participants faces and shoulders from either the adolescent or parent's perspective. The estimated 60-degree field of view occasionally resulted in participants being cut out of view, especially during movement. Tilted heads and focus on the Jenga tower caused participants faces to be out of view. The Charades game videos had relatively poor quality due to participant movement but faces remained visible as the game required eye contact. The Matching pairs card game showed less movement and better video quality due to participants being fixed on the cards on the table. The room lighting conditions for all game tasks were average, resulting in slightly dark videos and light reflections on faces. Due to the file size of a long continuous recording, the Teencams automated functionality was to split the video into multiple shorter 10-min videos for each viewpoint, which was time-consuming when saving and labelling the videos.

### 4.5. Understanding of the game

According to the researcher fieldnotes and the yes/no classification, differences were observed in the way the pairs understood the game. During the Matching pairs card game, parents understood the game the first time it was explained to them by the adolescent. Only one adolescent had to repeat the instructions of the game to their parent. For the Jenga game, it was observed that two adolescents did not understand the rules of the Jenga game from the researcher and therefore communicated the incorrect instructions to the parent. For those adolescents who correctly explained the instructions, it still took two teachings for three different parents to understand the instruction. Moreover, at some points, three parents needed the adolescents to repeat or remind them of the instruction. During the Charades games, all the adolescents could explain the instructions of the game properly and the parents understood the instruction at the first teaching. It was found that one parent participant took at least two teachings to understand the game across all workshops that were attended.

### 4.6. Quality of the interaction

The researchers observed variations in interaction quality among the pairs during the different games played across the three workshops. The Charades and Matching pairs card games fostered active engagement and stimulated game-related conversations, even leading to occasional accusations of cheating and increased competition between pairs. In contrast, the Jenga game elicited minimal to no interaction, with most pairs remaining quiet and focused to prevent the tower from collapsing.

### 4.7. Evaluation of the games for further analysis

The researchers evaluated all games collectively on the understanding of the game, audio, video, interaction quality and attendance. The Matching pairs card game was well understood by all participants and the instructions of the game were correctly followed. The Matching pairs card game also had the best audio quality due to the turn-taking of the game and the least amount of background noise from the other participants in the room. The video quality was also good for the Matching pairs card game as there was minimal movement and participants were hardly cut out of view. The interaction was also good as the game elicited competitiveness and topics of conversation that related to the game. Given that the game took place during the first workshop, attendance and participation were excellent. Therefore, the protocol for the BEACON cohort included the Matching pairs card game which was selected to be implemented in study 2 to inform research question 3.

## 5. Study 1: acceptability of the Teencam methodology (Phase 2)

### 5.1. Materials

#### 5.1.1. Interview schedule

The interview schedule focused on the acceptability of the Teencams and the feasibility of their use, from the perspectives of parents who took part in Phase 1. Parents were asked to report on any realized or foreseeable barriers to using the Teencams and any concerns they had. They were also asked to reflect on how they felt about being recorded, the comfort of the Teencams, whether they and their adolescent acted naturally during the observations and the game they enjoyed the most.

#### 5.1.2. Procedure

All parents who took part in Phase 1, were invited to return to the research site and take part in individual in-depth interviews. The individual interviews were facilitated by qualified research assistants who were proficient in English and also proficient in the local languages of the community (i.e., isiZulu). Interviews were audio recorded and ranged in length between 11 and 23 min. The audio recordings were transcribed verbatim and translated into English by the same qualified research assistants.

#### 5.1.3. Data analysis

To analyse the individual interviews with parents, ATLAS.ti version 8 was used to code and analyse the data using categorical content analysis (26). Analysis of the interview transcripts began with a period of reflection and internalisation of the data by the author FL. Preliminary codes were developed and defined based on the objectives of this Phase and emerging patterns from the transcripts. Codes were further refined after discussions with the remaining authors. These final codes were used to develop overarching themes. Illustrative quotes for each theme were extracted and presented in this manuscript (27).

## 6. Results

Of the 15 parents that were invited to participate in the one-on-one interviews, one parent passed away leaving 14 one-on-one interviews with parents. Interviews with parents on the acceptability of the Teencam methodology yielded the following themes: naturalistic behaviour, observational activity, and Teencam methodology for future research.

### 6.1. Naturalistic behaviour

During the first workshop, some parents (*n* = 9) reported feeling nervous, and self-conscious about being video recorded and claimed that this initially impacted their and their adolescent's ability to behave naturally. However, by the third workshop, participants were familiar with the methodology, began to feel more comfortable being recorded and behaved more naturally. Some participants felt that they acted naturally throughout all the workshops, but others stated that they were always aware of being recorded and held back negative behaviour. Some parents (*n* = 4) felt that the placement of the head camera was uncomfortable causing irritation, limiting their movement and leading them to always be aware of the camera recording.

*“At first I felt like I should be careful of whatever that I do or say but as time went by I was free.” (BAG 13, 46 year old parent)*

*“I’m always myself, if I want to reprimand my child I will do so while wearing the cameras. I won*’*t lie about who I am to impress you guys.” (BAG 11, 33 year old parent)*

*“…she kept on saying that mum don*’*t say some things because they can hear everything that we are saying.” (BAG 9, 56 year old parent)*

*It*’*s not comfortable, it*’*s disturbing, you can*’*t open your eyes. You must focus on it, basically, it must always be in the right space and not move.” (BAG 7, 34 year old parent)*

### 6.2. Observational activities

The most enjoyable observational task was the Matching pairs card game and Jenga. In particular, parents stated that they were familiar with the Matching pairs card game and often play it with their children at home. Parents reported Charades being the least enjoyable game as it allowed more opportunities for cheating and the game relied on the ability of the opponent to demonstrate well.

*“It*’*s the same cards that we usually play in the house. It*’*s quite easy to understand, It*’*s not complex.” (BAG 2, 29 years old parent)*

Almost all parents (*n* = 11) preferred that the adolescent instruct and teach the parents the game as it was a valuable educational exercise for their adolescent. One parent felt that adolescents had an unfair advantage because they were already familiar with the game. Others suggested a rotation among parents and adolescents in teaching and instructing each other.

*“It*’*s a good idea because it shows that she learnt what she was doing with you. If she can teach me, it shows that she was listening.” (BAG 15, 37 year old parent)*

*“I also wanted to win, It*’*s not fair. The adolescents were winning because they were taught the game before.” (BAG 11, 33 year old parent)*

*“Maybe you can alternate, have the parents teach the kids this week and the kids teach the parents the following week” (BAG 2, 29 year old parent)*

### 6.3. Teencam methodology for future research

When parents were asked about their overall impressions of the Teencam methodology, almost all participants had positive views. Parents highlighted that it allowed them to communicate with their children and one parent was willing to take the head cameras home for further observation. On the other hand, one participant was concerned about confidentiality and the way the videos were going to be used. Another participant felt that the Teencam was too intrusive and preferred to talk to researchers rather than be video recorded.

*“Teencam has given me the chance to communicate with my child, I wish we could take it home” (BAG 3, 41 year old parent)*

*“I don*’*t have a problem with Teencam, I was only concerned about who is going to watch the footage.” (BAG 6 & 16, 37 year old parent)*.

*“I don*’*t like Teencam, I feel like if you guys want to know us you can… I think you can know us better through communication instead of putting cameras on our foreheads.” (BAG 11, 33 year old parent)*

Most parents (*n* = 10) also stated that future participants would be willing to participate in the Teencam methodology, provided that the researchers orientate and reassure participants on the purpose, privacy and use of the video footage.

*“I think they will be willing as long as it is explained from the onset that it is not played for the other people.” (BAG 2, 29 year old parent)*

## 7. Study 2: codability of automated software

Study 2 addressed objective 3 and consists of one phase that explored the codability of the videos using automated coding software.

### 7.1. Research participants

Phase 2 consisted of a Respondent Driven Sampling (RDS) approach (28) to recruit participants into the BEACON cohort. RDS used in previous surveys in South Africa (29–31) uses a chain referral system that begins with a purposefully selected convenience sample or “seed”. The seeds in this study came from the following sources: (1) BAG parents and (2) community recruiters who were responsible for recruiting eligible participants but did not participate in the study. Each seed was required to identify and refer eligible participants to the study and these individuals in turn referred potential participants to the study, and so on. This procedure creates an expanding system of chain referrals characterized by “waves” of recruitment. Unlike snowball sampling, RDS produces more reliable data estimates by using a “link-tracing design” that estimates participant network sizes and calculates selection probabilities between recruiters and their recruits (32). Through a sufficient number of waves of participant recruitment, a bias that the non-random choice of seeds may have introduced is overcome, which stabilises the composition of the sample, thereby becoming independent of the seeds from which recruitment began (33).

Survey administrators made use of an electronic system to help track the RDS recruitment chain. Recruits interested in participating in the study had the option of (1) Submitting their contact numbers through an electronic link sent to them via text message, which allowed an online screening eligibility survey to be sent to them at no data cost. (2) Submitting their contact numbers via WhatsApp and having a survey administrator call the participant to complete the screening eligibility survey over the phone. (3) Have their recruiters forward them a screening link which they completed and submitted with their recruiter. For every successful recruit, the recruiter received a monetary incentive (ZAR 10) (USD 0.54).

### 7.2. Procedure

**Figure 3:**
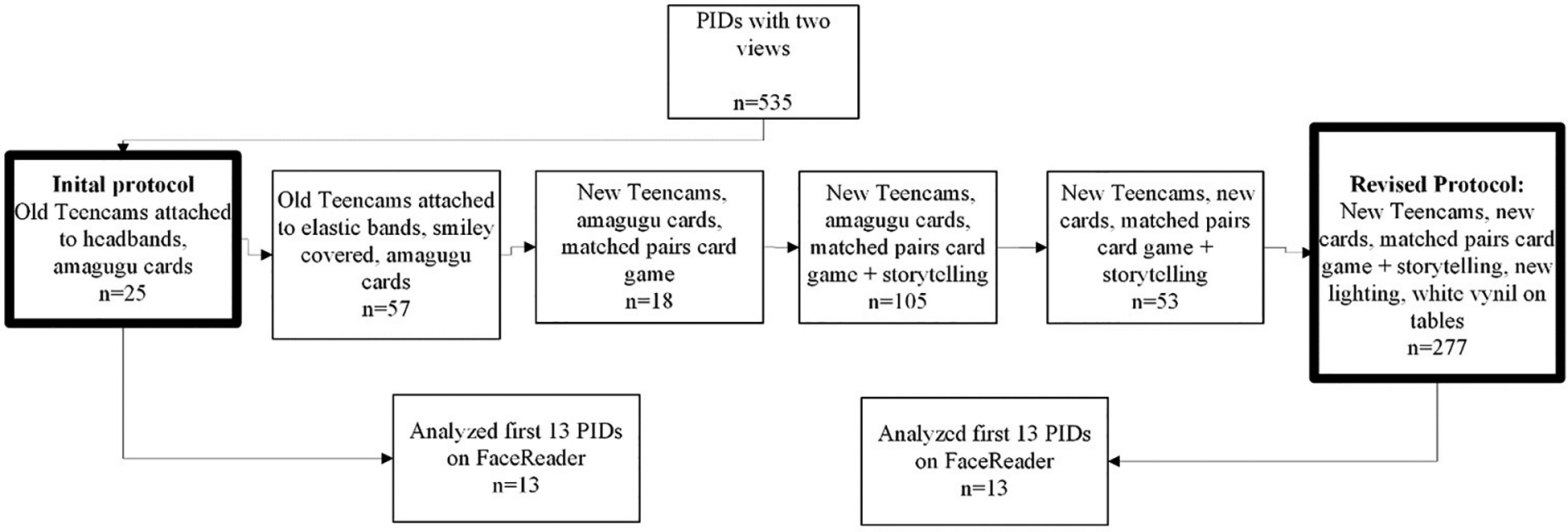
Timeline of improvements to improve video quality and FaceReader output.

This study involved testing the methodological protocol developed in study 1 on additional parent-adolescent pairs who were enrolled in the BEACON cohort. The purpose was to assess the effectiveness of automated coding software, FaceReader, in analyzing the video clips. FaceReader is a fully automated facial recognition software capable of coding six basic emotions: happiness, sadness, surprise, fear, disgust, and anger (www.noldus.com/facereader).Videos for analysis were chosen consecutively and had to include recordings from both the parent and the adolescent. The protocol underwent enhancements and refinements to improve FaceReader's ability to detect and code facial expressions. Following each enhancement, the output from FaceReader was recorded to identify any improvements in the automated coding of emotions through facial expressions. Following several enhancements, a final methodological protocol was developed. Figure 3 illustrates a timeline of the refinements made.

Video clips before the refinements had the following protocol (initial protocol): As in phase 1, videos were recorded using the Teencams with a yellow lapel badge and parent-adolescent pairs played the Matching pairs card game using the Amagugu cards (*n* = 25). The Teencams would automatically split the video footage of the observation into 10-min video clips resulting in two video clips for each viewpoint. The lighting conditions of the assessment room contained a single fluorescent light in the middle of the room and windows on a single side that allowed for natural light to enter the room.

Refinements leading to the final revised protocol: It was found that the smiley faces on the Teencam were being coded, rather than the participant's face and the headband that the camera was attached to was obstructing the eyebrows, preventing FaceReader from detecting facial expressions. The cameras were then attached to a thin elastic band and the smiley faces on the Teencams were covered (*n* = 57) but this resulted in only marginal improvements. The limitations of movements and narrow-angle views would still cut out the participant's face.

**Figure 4:**
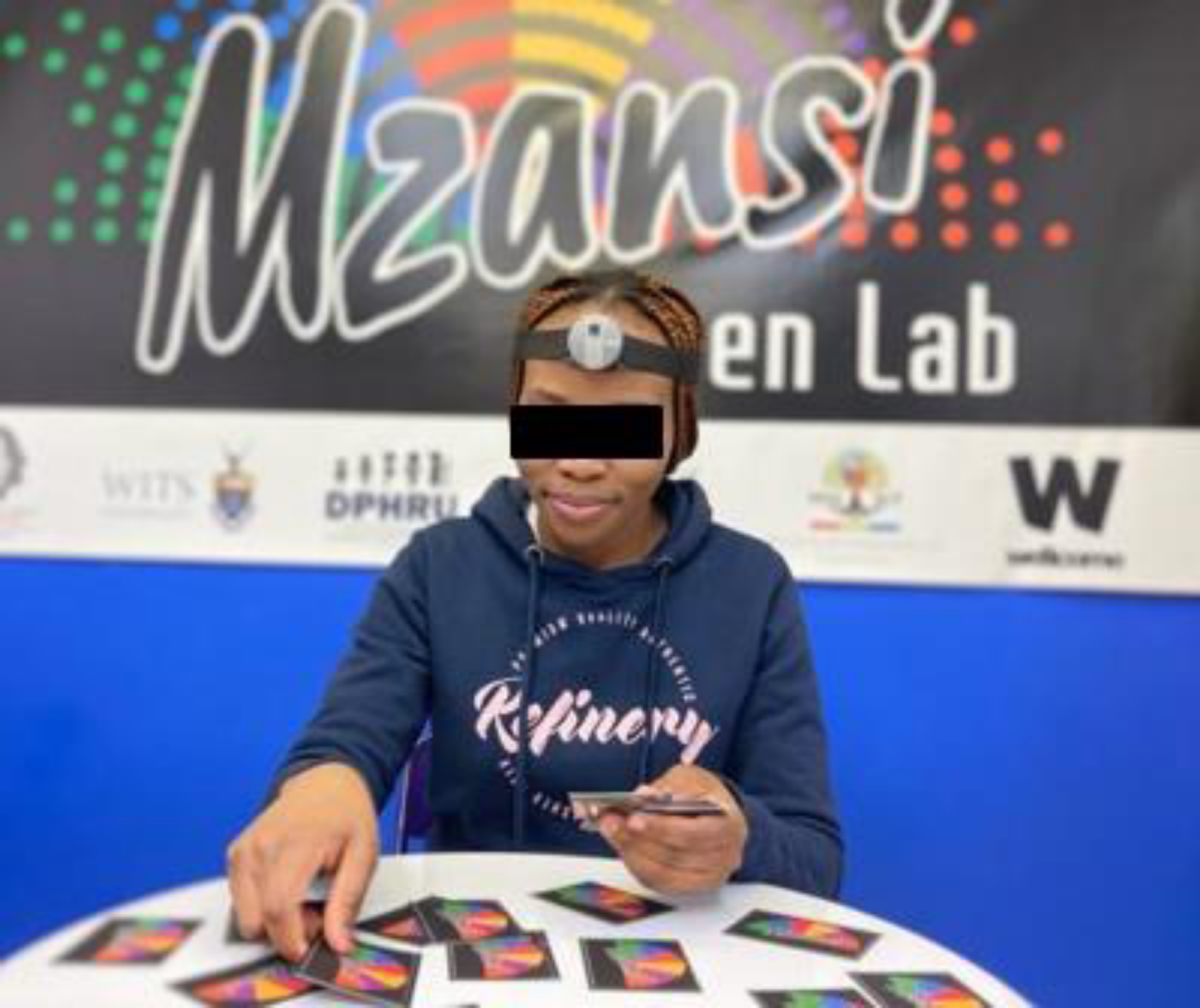
Parent view of improved video quality.

Findings from the feasibility and acceptability phase of the study, as well as the codability of the videos, called for refinements and adjustments to the head cameras. New head cameras were used and had the following improvements: Head cameras were lightweight and attached to a thin and adjustable headband that did not obstruct the participants’ eyebrows, allowing the detection of facial expressions. A blue light was visible to indicate when cameras were switched on and recording. Cameras included improved video resolution with a wide-angle view which is necessary for FaceReader to capture the facial expressions and muscles needed to code (180 degrees). One video clip for the entire observation from each viewpoint was also recorded. The cameras were grey and did not contain any facial features (*n* = 18) (see Figure 4).

To increase interaction and communication between the parent-adolescent pairs, the Matching pairs card game was further refined to make the cards more relatable and was called Teentalk. Teentalk consists of Matching card pairs designed by the researchers that consisted of eight cards with images displaying positive behaviours (peers playing and learning, positive parent-adolescent interactions and adolescents using technology) and seven cards with images displaying negative behaviours (adolescents using alcohol and tobacco, harsh parenting, bullying, adolescents looking depressed, teenage pregnancy). The Teentalk card game followed the same instructions as the Matching pairs card game. At the end of the game, participants were instructed to shuffle and distribute the cards equally amongst each other and then hold up a card from that pile and tell a story.

The lighting conditions were further improved by including more fluorescent lighting from the ceiling, white tables and open curtains to allow for natural light from the side of the room. These adjustments in the lighting illuminated the participant's face from nearly all angles and eliminated shadows as seen in Figure 4. Although individual booths were considered to minimize the background noise from other participants, there were spacing and lighting issues that also came up.

### 7.3. Participant exclusions and final sample

The primary aim of the BEACON cohort study is to investigate the relationship between conduct disorder and executive function (EF). A final analytic sample of *n* = 640 will provide >80% power (alpha 0.05) to detect a 6% difference (which is considered clinically relevant) in conduct disorders across equal size grouped binary or continuous EF composite scores, with power to detect correlations of 0.7 (medium effect size delta 0.05).

715 parent-adolescent pairs were enrolled in the BEACON study through RDS approaches. However, 129 pairs did not record videos due to technical difficulties and time constraints, 2 pairs recorded their videos in a different room due to construction sounds in the designated room. 40 pairs did not have the complete questionnaire data required by the BEACON study and 9 parent-adolescent pairs had recordings with either only the adolescent's view or the parent's view. The final sample of 535 parent-adolescent videos was achieved. The first 13 parent-adolescent pairs were selected from the group using the initial protocol and their FaceReader output was compared to the group employing the revised protocol (*n* = 13) as seen in Figure 3.

#### 7.3.1. Participant characteristics

In total, there were 535 parent-adolescent pairs in this study as presented in Table 3. There was almost an equal gender distribution of adolescents but more mothers (93.46%) were enrolled compared to fathers. The average age of the adolescents was 11.88 years (SD = 0.59) and the average age of parents was 38.67 years (SD = 7.39). Most pairs identified themselves as African (98.69%). Only a quarter of parents were employed (24.67%) and half the parents reported to have obtained matric (senior school certificate) qualification.

**Table 3:** Participant characteristics of the BEACON participants.

	Adolescent		Parent	
	*n*	%/mean (SD)	*n*	%/mean (SD)
Age	535	11.88 (0.59)	535	38.67 (7.39)
Gender				
Male	257	48.04	35	6.54
Female	278	51.96	500	93.46
Race				
African			528	98.69
Coloured			7	1.31
Employed			132	24.67
Highest education level				
None			17	3.18
Primary school			124	23.22
Matric			265	49.63
Post-matric			59	11.05
Other			69	12.92

### 7.4. Data analysis

This study used FaceReader version 9, issued by Noldus (www.noldus.com/facereader), to automatically analyse the video data of the observations. FaceReader automatically analyses the video every 0.033 s and produces an analysis on (1) whether the face was detected and analysed on any of the seven basic facial expressions (happy, sad, surprise, disgust, angry, fear, neutral) (2) the face was detected but could not be analysed or (3) no face was detected. The intensity of a facial expression detected was recorded on a scale between 0 (no intensity) to 1 (high intensity). FaceReader output was analysed by investigating the proportion of time that FaceReader could detect and code a face and the number of times FaceReader was able to code facial expressions. An independent samples *t*-test was used to detect significant differences in the software's ability to detect and code the faces between the initial and revised protocol group.

### 7.5. Results

The detection and codability significantly improved in the revised protocol (mean = 32.803; SE = 1,940) compared to the initial protocol (mean = 11.885; SE = 859), as indicated by a substantial increase. This difference was found to be highly significant: *t* (24) = −9.86, *p* < 0.001). Furthermore, the revised protocol demonstrated a significantly longer average duration of detection (mean = 18.22; SE = 1.1) compared to the initial protocol (mean = 6.6; SE = 0.48), with a similar level of statistical significance: *t* (24) = −9.86, *p* < 0.001.

## 8. Discussion

This study aimed to explore the acceptability and feasibility of using a video observational methodology to observe parent-adolescent communication and interaction using wearable headcams in an urban setting in South Africa. To our knowledge, no protocols have been developed for objectively measuring parent-adolescent interactions in urban South African settings. Little is known about how to code these data within the South African cultural and socio-economic context and how acceptable these observational methods may be for the parent or adolescent participants in this context.

This study found that the use of head cameras to capture parent-adolescent interactions was an acceptable and feasible method. This finding is in line with another study that recorded infant-mother interactions in the same context (22). The head cameras were able to successfully capture parent-adolescent communication and interaction, although there were some challenges with the technical operations of the head cameras. Studies using similar head cameras to capture mother-infant views also had challenges in turning the camera on and off and headcam placement (22, 23). While this study made improvements on the camera, the placement was still a challenge as indicated by the feedback received from parents. As found in a South African study recording infant-mother interactions, the placement and presence of the headcam still made some participants conscious of their behaviour, promoting socially desirable behaviours and suppressing inappropriate behaviours (22). However, in line with other studies using the headcam, the majority of the participants reported not being aware of the camera recording which increased their likelihood of natural behaviour (18, 22, 23). These findings show that although camera awareness was not eliminated, this method of capturing behaviour is still an improvement from having a researcher present in observing the interaction.

Literature on parent-child interaction has been examined in the context of mealtime (34–37), play-interactions (38), and parent-child conflict discussions (39). This study builds on existing literature by testing the feasibility of measuring parent-adolescent communication and interactions using game interactions. The feasibility of three different popular games (Matching pairs card game, Jenga, Charades) were tested while wearing head cameras to record video and audio of participant viewpoints while completing the game. This study found that the Matching pairs card game was able to elicit prosocial behaviour, competitiveness, problem solving, conflict resolution and communication skills while recording participant viewpoints. Similar to the literature that looks at parent-child interactions in the context of mealtime, the Matching pairs card game gave researchers an insight into the quality of the parent-child relationship that was stimulated over a game interaction. Therefore, studying dyadic game interactions offers an event to better understand parent-adolescent communication and interaction.

This study also further refined the Matching pairs card game to include custom-designed playing cards called “Teentalk”. The “Teentalk” cards displayed images of risk (pregnancy, smoking, alcohol use, bullying, depression, harsh parenting), protective (friendship, green spaces, positive parenting) and digital (adolescents on electronic devices in different locations) behaviours. Parent-adolescent pairs used the “Teentalk” cards in a storytelling game, which gave insight into the way parents and adolescents talk about risk and protective behaviours while capturing video footage of their moods and emotion. According to the literature, there is a strong indication that open parent-child communication influences the reduction of risky behaviours among adolescents, such as substance use and abuse, delinquent behaviours and risky sexual behaviours (40, 41). Research conducted in South Africa is limited and has only focused on parent-adolescent communication in the context of its association with sexual risky behaviours and reproductive health (42) sexuality and HIV/AIDS (5, 43). This study offers in-depth rich contextual data on barriers and protective factors in parent-child communication from the perspectives of both parents and adolescents. This could lead to a better understanding of how parent-adolescent relationships can be supported through effective communication and interaction to minimise adolescent risk and enhance resilience.

Finally, this study revealed that the utilization of an advanced coding software called FaceReader (https://www.noldus.com/facereader) was able to automate the coding process of facial expressions. The coding software however requires very specific conditions: participants’ faces need to be in view with eyebrows visible, no faces in the background only the participant should be visible, movement should be limited and the environment should be well-lit. While this coding software was able to successfully detect facial expressions of both parents and adolescents there were instances where faces could not be detected due to adolescents looking away during conversation or hand gestures blocking the view of the face. These behaviours that could not be detected by FaceReader may still be important, especially with adolescents. The looking away may be an indication of respect, especially from the adolescents since in the African culture or tradition, maintaining eye contact for too long with adults may be seen as disrespectful (44), and the hand gestures may signify boredom. Here it may be still valuable for researchers to manually code these instances possibly using micro-coding tools as presented in these studies (18, 23). Nonetheless, this coding software has the potential to still strengthen methodological approaches in contexts like South Africa, with fewer resources and less time needed to collect and score objective data.

A limitation of the study includes observations taking place in a “controlled” environment with a researcher facilitating the process, although the researcher left the room for interactions to occur and only came back at the end of the recording time to switch off the cameras. It is recommended that future researchers consider having their pairs take the Teencams home to record their interactions in the home environment and to measure parent-adolescent communication and interaction in a natural setting. Our objective was to gather feedback on the acceptability of the Teencam methodology by conducting interviews with both adolescents and parents during workshop three. However, due to time constraints and the adolescents’ fatigue from completing other assessments as part of the BEACON study, the researcher made the decision to interview only the parent participants. Future studies should consider gaining adolescent perspectives as well.

## 9. Conclusion

This study showed that using Teencam video observational methodology to measure parent-adolescent communication and interactions is acceptable and feasible in Soweto, South Africa. The unique contribution of this research lies in its potential to lead to improved methodologies for measuring parent-adolescent communication and interactions. In time, this pilot study could lead to innovations in our understanding of how to support parenting practices and adolescent development in high-risk contexts. Particular observational protocols however do need to be adapted for the software to successfully detect the faces and code the facial expressions and emotional cues. These include room and lighting conditions, eye contact, movement and the quality of the device. Important gestures that could not be detected by the software could be micro-coded by the researcher using available software.

## Data Availability

The raw data supporting the conclusions of this article will be made available by the authors, without undue reservation.
